# Epidemiology of Angioid Streaks and Pseudoxanthoma Elasticum (2011–2020): A Nationwide Population-based Cohort Study

**DOI:** 10.1016/j.xops.2023.100370

**Published:** 2023-07-20

**Authors:** Saori Wada, Masahiro Miyake, Ai Kido, Takuro Kamei, Shusuke Hiragi, Hanako Ohashi Ikeda, Masayuki Hata, Hiroaki Ueshima, Akitaka Tsujikawa, Hiroshi Tamura

**Affiliations:** 1 Department of Ophthalmology and Visual Sciences, Kyoto University Graduate School of Medicine, Kyoto, Japan; 2 Kyoto Okamoto Memorial Hospital, Kyoto, Japan; 3 Medical Research Institute KITANO HOSPITAL, PIIF Tazuke-kofukai, Osaka, Japan; 4 Center for Innovative Research and Education in Data Science, Institute for Liberal Arts and Sciences, Kyoto University, Kyoto, Japan

**Keywords:** Angioid streaks, Pseudoxanthoma elasticum, Epidemiology, Incidence, Prevalence

## Abstract

**Purpose:**

We aimed to describe the epidemiology of angioid streaks (AS) and pseudoxanthoma elasticum (PXE), which are rare diseases, using a national claims database.

**Design:**

This was a population-based longitudinal cohort study.

**Participants:**

A total of 126 million individuals were covered by the universal health coverage system in Japan.

**Methods:**

With permission from the Ministry of Health, Labor and Welfare, we accessed all data from the National Database of Health Insurance Claims and Specific Health Checkups of Japan, which contains the nationwide health insurance claims data for 126 million Japanese. We identified individuals with AS and PXE between January 2011 and December 2020. The incidence rates, prevalence, overlap of AS and PXE, and mean age at death were calculated.

**Main Outcome Measures:**

The incidence rates and prevalence of AS and PXE.

**Results:**

A total of 6598 cases of AS and 1020 cases of PXE were identified during the 10-year study period. The incidence rates of AS and PXE were 0.52 (95% confidence interval, 0.48–0.56) and 0.08 (95% confidence interval, 0.07–0.10) per 100 000 person-years, respectively. On October 1, 2020, the prevalence of AS and PXE was 6.5 (95% confidence interval, 6.38–6.66) and 0.83 (95% confidence interval, 0.78–0.89) per 100 000 persons, respectively. The overlap of AS and PXE was 363 patients. The mean age at death of individuals with AS and PXE was 79.3 ± 0.51 and 77.1 ± 2.68 years, respectively.

**Conclusion:**

This is the first population-based study to elucidate the epidemiology of AS and PXE. The mean age of death of both AS and PXE patients was younger than the mean life expectancy of the general Japanese population, thus, appropriate diagnosis and management are important to avoid preventable death.

**Financial Disclosure(s):**

Proprietary or commercial disclosure may be found in the Footnotes and Disclosures at the end of this article.

Angioid streaks (AS), first described by Doyne in 1889,^1^ are characterized by breaks in the Bruch’s membrane radiating from the optic disc that are often bilateral.^2^, ^3^, ^4^ Angioid streaks result from calcification of Bruch’s membrane. When associated with macular neovascularization,^2^^,^^5^, ^6^, ^7^ AS are often treatment-resistant and can progress to poor vision and blindness.^8^ Pseudoxanthoma elasticum (PXE), described by Rigil in 1881,^9^ is an autosomal recessive disease characterized by dystrophic calcification of elastic fibers throughout the cutaneous, ocular, cardiovascular tissues, and other manifestations.^10^, ^11^, ^12^ Grönblad and Strandberg highlighted the close relationship between AS and PXE, therefore, AS and PXE are often considered a set,^13^^,^^14^ and together referred to as Grönblad–Strandberg syndrome.^9^ It has been reported that 59% to 87% of PXE cases are accompanied by AS,^3^ but the actual situation is unclear because most evidence is based on small-scale surveys or simulations.

Pseudoxanthoma elasticum is comorbid with potentially life-threatening complications, including a 3.6-fold increase in the relative risk of ischemic stroke, hemorrhage of the gastrointestinal or urinary tract, and pulmonary dysfunction.^2^^,^^9^ Especially for patients > 50 years old, the relative risks of ischemic heart disease and cerebral infarction are up to 5.5 and 34, respectively.^15^ Additionally, most quality of life loss in PXE is due to visual impairment caused by macular neovascularization in AS.^6^^,^^16^ As evidenced by the ongoing industry-sponsored clinical trial (available at https://jrct.niph.go.jp/latest-detail/jRCT2071220090, accessed February 24, 2023) to establish anti-VEGF therapy, faricimab, as one of the main treatment for macular neovascularization associated with AS, it is recognized as a medically and commercially significant disease. Despite the clinical importance of AS and PXE, their epidemiological information is limited because they are rare diseases. For the better management of AS and PXE, evidence-based information about their incidence and prevalence is required.

To uncover the epidemiology of rare diseases, a nationwide population-based cohort study is required. For this purpose, the National Database of Health Insurance Claims and Specific Health Checkups of Japan (NDB) would be the most applicable. This is one of the largest claims databases in the world, managed by the Japanese national government.^17^, ^18^, ^19^, ^20^, ^21^, ^22^, ^23^ Recently, considerable epidemiological evidence has been revealed using the database.^17^, ^18^, ^19^^,^^24^, ^25^, ^26^ For example, we recently reported the epidemiology of central serous chorioretinopathy,^17^ age-related macular degeneration,^19^ and central retinal artery occlusion.^18^ Hashimoto et al^24^ also clarified the incidence of sympathetic ophthalmia after inciting events using the database. As such, NDB is accepted as a reliable and useful database due to its comprehensive coverage and scale.

In the current study, we accessed and analyzed all data stored in the NDB with the permission of the Japanese Ministry of Health, Labor and Welfare (MHLW) to clarify the epidemiological background of AS and PXE. This study revealed the incidence and prevalence of clinically-manifested AS and PXE, the proportion of their overlap, and the age at death of patients with these diseases.

## Methods

We conducted a retrospective cohort study using the NDB, containing health insurance claims data of almost all (≥ 95%) medical treatments of 126 million individuals in Japan.^17^, ^18^, ^19^, ^20^, ^21^, ^22^^,^^27^ This retrospective, nationwide population-based cohort study was approved by the institutional review board and the ethics committee of Kyoto University Hospital and Kyoto University Graduate School of Medicine (No. R2405-1). All investigations adhered to the tenets of the Declaration of Helsinki. The need for informed consent was waived because of the use of legally anonymized data.

### Database

Although detailed information on the NDB is described in our previous reports,^17^, ^18^, ^19^ we briefly explain it here.

The universal health insurance system in Japan covers the entire population of 126 million individuals. All claims data are principally submitted electronically and stored in the NDB, administered by the MHLW from 2011.^17^^,^^19^, ^20^, ^21^, ^22^ The data can be accessed through the NDB Onsite Research Center, Kyoto, which is 1 of 2 NDB onsite remote access centers with access to the entire NDB dataset, with the approval of the MHLW.^22^ The current study was conducted during the approved study period from August 23, 2021 to February 22, 2022.

The NDB contains information on medical claims data in Japan, including diagnoses coded according to the *International Statistical Classification of Diseases and Related Health Problems, Tenth Revision* (ICD-10), drugs, and inpatient and outpatient procedures. These are also coded with local claim codes in Japan, including the 7-digit local diagnostic codes (“NDB diagnostic codes,” hereafter), which are more specific than ICD-10 codes.^17^, ^18^, ^19^ When the research was initiated, data on a total of > 14 billion claims were available for the entire Japanese population (n ≥ 126 million) generated between 2011 and 2020.

### Identification of AS and PXE

After linking all claims data of individuals as previously reported,^17^^,^^23^ we identified patients who were diagnosed with AS and PXE, defined by the NDB diagnostic codes of AS (NDB diagnostic code “8840626,” ICD-10 code H35.3) and PXE (NDB diagnostic code “8848612,” ICD-10 code Q82.8) at any time between January 1, 2011, and December 31, 2020.

### Incidence and Prevalence of AS and PXE

The onset of AS and PXE was defined as when the date of their diagnoses matched the month in which their disease names were first assigned to the claims data. The number of cases of AS and PXE onset, namely the incidence between 2011 and 2020, and the number of living patients, namely the prevalence, as of October 1, 2020, of AS and PXE, were counted by age and sex. The incidence rates during the study period and the prevalence stratified by age and sex were determined by dividing the number of AS and PXE cases within each group by the population at risk within the corresponding group.^17^, ^18^, ^19^ The age-standardized incidence rates and prevalence of AS and PXE were calculated based on the standard age-structure world population of the World Health Organization for 2000 to 2025.^28^ The current population estimates of each year between 2011 and 2020 provided by the Japanese Ministry of Internal Affairs and Communications were used to define the entire population and each subgroup population as the population at risk (available at https://www.stat.go.jp/english/data/jinsui/2.html, accessed September 10, 2022).

### Overlapping of AS and PXE

To evaluate the comorbidity between AS and PXE, we evaluated the overlap of both diseases. Cases as of October 1, 2020, in which the same patient had both NDB diagnostic codes for AS and PXE were defined as overlapping cases of AS and PXE and counted. Whether AS or PXE was diagnosed first or in the same month was also evaluated. The proportion of overlaps within each case of AS and PXE was calculated.

### Number of Deaths and Mean Age at Death in Individuals with AS and PXE

To infer the life expectancy of the patients with AS and PXE, we evaluated the age of death of these patients. For this analysis, individuals whose deaths were explicitly indicated in the claims data were considered to have died on that date. The number of AS and PXE patients who died by October 1, 2020, was counted by age. Their mean age at the time of death was also calculated, to evaluate the life expectancy of patients with these life-threatening diseases.

### Statistical Analyses

All statistical analyses were performed using Oracle R Enterprise 1.4.1 (Oracle Corporation) and R version 3.4.1 (R Foundation for Statistical Computing). All values are presented in 95% confidence intervals based on the Poisson distribution. The MHLW prohibits the publication of data that contains < 10 patients within each group in NDB utilization studies to protect personal information.^29^ We reported the data in compliance with the rules, after attempting to merge several groups. Z-test was used to compare the mean age of death for AS and PXE with the mean life expectancy of the general Japanese population. Two-sided *P*-values < 0.05 were considered statistically significant.

## Result

**Figure 1:**
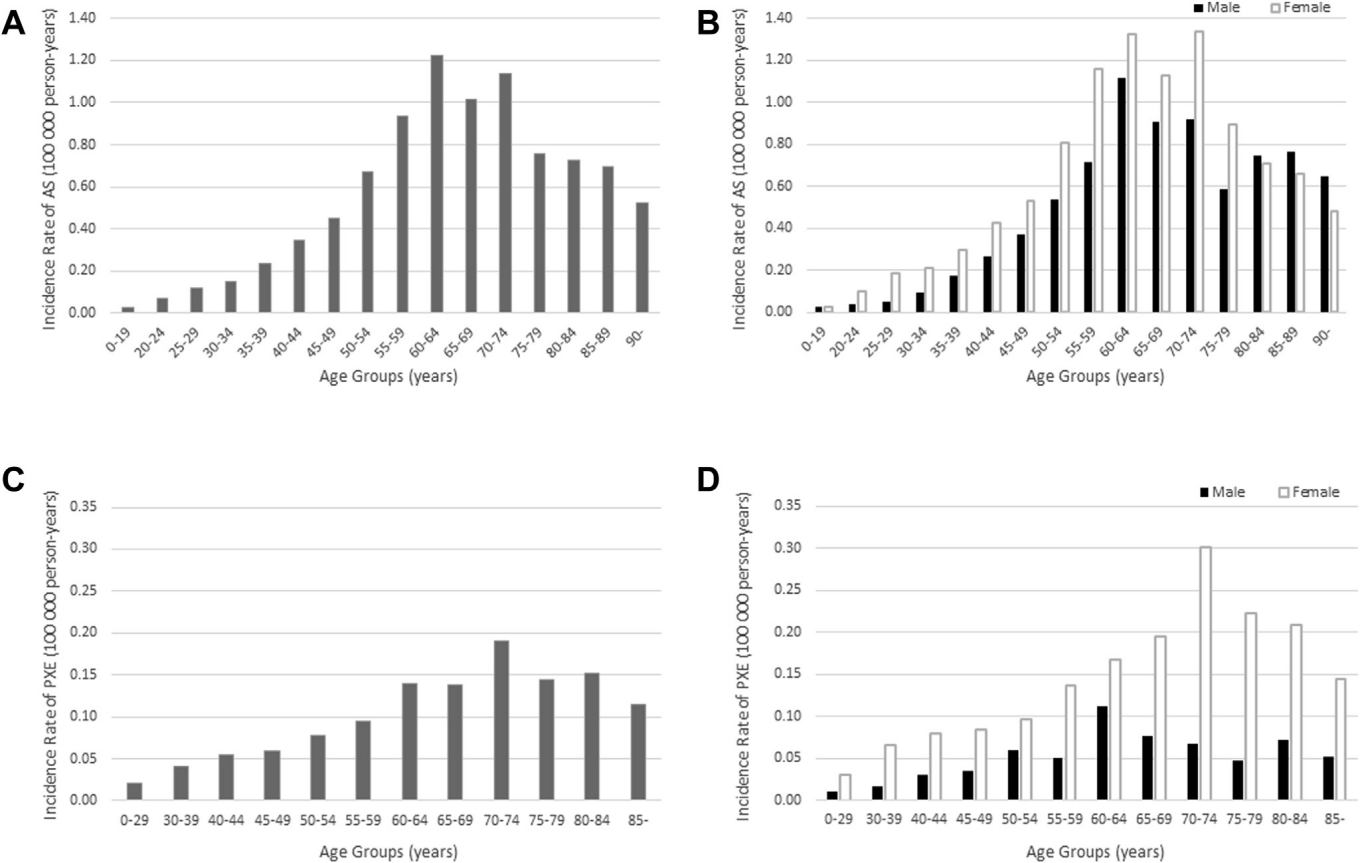
The incidence of angioid streaks (AS) and pseudoxanthoma elasticum (PXE). The age-stratified incidence rate of AS (**A**) and the age- and sex-stratified incidence rate of AS (**B**) are presented. Overall, a bimodal peak was observed in the 60 to 64 and 70 to 74 age groups. By sex, bimodal peaks were observed in males aged 60 to 64 and 85 to 89 years, and in females aged 60 to 64 and 70 to 74 years. The second peak occurred later in males than in females. These bimodalities may reflect the phenotypic variation of AS. Similarly, the age-stratified incidence rate of PXE (**C**) and the age- and sex-stratified incidence rate of PXE (**D**) are presented. Overall, a peak was observed in the 70 to 74 age group without any bimodal peaks as seen in AS. By sex, a bimodal peak was observed in males aged 60 to 64 and 80 to 84 years, while in females, a peak was observed in the 70 to 74 age group.

Figure 1 and Tables 1 and 2 show the age-stratified and sex-stratified incidences and incidence rate of AS and PXE. In total, 6598 cases of AS were identified in the NDB dataset during the 10-year study period, among which 60.2% were female. The incidence rate of AS was 0.52 per 100 000 person-years for the overall population, which corresponded to 0.30 per 100 000 person-years after age standardization to the World Health Organization’s standard world population. The incidence rate of AS was higher in females than males up to 80 years old, and higher in males than females > 80 years old. The highest incidence rate was observed in males aged 60 to 64 years and females aged 70 to 74 years. In total, 1020 cases of PXE were identified in the NDB dataset during the 10-year study period, among which 75.7% were female. The incidence rate of PXE was 0.08 per 100 000 person-years for the total population, which corresponded to 0.05 per 100 000 person-years after age standardization to the standard world population from the World Health Organization. The incidence rate of PXE was higher in females than males in all age groups. The highest incidence rate was observed in males aged 60 to 64 years and females aged 70 to 74 years.

**Table 1:** The Age-stratified and Sex-stratified Incidences and Incidence Rate of Angioid Streaks in Japan from 2011 to 2020, as Determined by the National Database of the Health Insurance Claims and Specific Health Checkups of Japan

Age Groups (Years)	Japanese Population∗			Incidence			Incidence Rate (95% CI)†		
	Male	Female	Total	Male	Female	Total	Male	Female	Total
0–19	10 619	10 113	20 731	25	24	49	0.02 (−0.01 to 0.05)	0.02 (−0.01 to 0.05)	0.02 (0–0.04)
20–24	3289	3081	6370	13	30	43	0.04 (−0.03 to 0.11)	0.10 (−0.01 to 0.21)	0.07 (0–0.13)
25–29	3241	3036	6277	17	56	73	0.05 (−0.03 to 0.13)	0.18 (0.03–0.34)	0.12 (0.03–0.2)
30–34	3371	3225	6596	31	68	99	0.09 (−0.01 to 0.19)	0.21 (0.05–0.37)	0.15 (0.06–0.24)
35–39	3756	3655	7411	66	109	175	0.18 (0.04–0.31)	0.30 (0.12–0.48)	0.24 (0.13–0.35)
40–44	4261	4157	8419	113	176	289	0.27 (0.11–0.42)	0.42 (0.23–0.62)	0.34 (0.22–0.47)
45–49	4951	4846	9797	182	258	440	0.37 (0.20–0.54)	0.53 (0.33–0.74)	0.45 (0.32–0.58)
50–54	4366	4318	8684	234	348	582	0.54 (0.32–0.75)	0.81 (0.54–1.07)	0.67 (0.50–0.84)
55–59	3932	3940	7872	280	455	735	0.71 (0.45–0.98)	1.15 (0.82–1.49)	0.93 (0.72–1.15)
60–64	3667	3764	7431	409	499	908	1.12 (0.77–1.46)	1.33 (0.96–1.69)	1.22 (0.97–1.47)
65–69	4015	4268	8283	363	480	843	0.90 (0.61–1.20)	1.12 (0.81–1.44)	1.02 (0.80–1.24)
70–74	4332	4846	9178	397	648	1045	0.92 (0.63–1.20)	1.34 (1.01–1.66)	1.14 (0.92–1.36)
75–79	3182	3939	7120	187	351	538	0.59 (0.32–0.85)	0.89 (0.60–1.19)	0.76 (0.55–0.96)
80–84	2248	3168	5416	167	225	392	0.74 (0.39–1.10)	0.71 (0.42–1.00)	0.72 (0.50–0.95)
85–89	1332	2403	3735	102	158	260	0.77 (0.30–1.24)	0.66 (0.33–0.98)	0.70 (0.43–0.96)
> 90	619	1813	2433	40	87	127	0.65 (0.01–1.28)	0.48 (0.16–0.80)	0.52 (0.23–0.81)
All patients	61 181	64 572	125 753	2626	3972	6598	0.42 (0.37–0.47)	0.62 (0.55–0.68)	0.52 (0.48–0.56)

∗ Japanese population data (as of October 1, 2020) were based on the Japanese Ministry of Internal Affairs and Communications. (Unit: 1000 people).

† The incidence rate is reported per 100 000 person-years. CI = confidence interval.

**Table 2:** The Age-stratified and Sex-stratified Incidences and Incidence Rate of Pseudoxanthoma Elasticum in Japan from 2011 to 2020, as Determined by the National Database of the Health Insurance Claims and Specific Health Checkups of Japan

Age Groups (Years)	Japanese Population∗			Incidence			Incidence Rate (95% CI)†		
	Male	Female	Total	Male	Female	Total	Male	Female	Total
0–29	17 149	16 230	33 378	18	50	68	0.01 (0–0.03)	0.03 (0–0.06)	0.02 (0.01–0.04)
30–39	7127	6880	14 007	12	45	57	0.02 (−0.01 to 0.05)	0.07 (0–0.13)	0.04 (0.01–0.07)
40–44	4261	4157	8419	13	33	46	0.03 (−0.02 to 0.08)	0.08 (−0.01 to 0.17)	0.05 (0–0.1)
45–49	4951	4846	9797	17	41	58	0.03 (−0.02 to 0.09)	0.08 (0–0.17)	0.06 (0.01–0.11)
50–54	4366	4318	8684	26	42	68	0.06 (−0.01 to 0.13)	0.10 (0–0.19)	0.08 (0.02–0.14)
55–59	3932	3940	7872	20	54	74	0.05 (−0.02 to 0.12)	0.14 (0.02–0.25)	0.09 (0.03–0.16)
60–64	3667	3764	7431	41	63	104	0.11 (0–0.22)	0.17 (0.04–0.30)	0.14 (0.05–0.23)
65–69	4015	4268	8283	31	83	114	0.08 (−0.01 to 0.16)	0.19 (0.06–0.33)	0.14 (0.06–0.22)
70–74	4332	4846	9178	29	146	175	0.07 (−0.01 to 0.14)	0.30 (0.15–0.46)	0.19 (0.10–0.28)
75–79	3182	3939	7120	15	88	103	0.05 (−0.03 to 0.12)	0.22 (0.08–0.37)	0.14 (0.06–0.23)
80–84	2248	3168	5416	16	66	82	0.07 (−0.04 to 0.18)	0.21 (0.05–0.37)	0.15 (0.05–0.26)
> 85	1951	4216	6168	10	61	71	0.05 (−0.05 to 0.15)	0.14 (0.33–0.26)	0.12 (0.03–0.20)
All patients	61 181	64 572	125 753	248	772	1020	0.04 (0.02–0.06)	0.12 (0.09–0.15)	0.08 (0.07–0.10)

∗ Japanese population data (as of October 1, 2020) were based on the Japanese Ministry of Internal Affairs and Communications. (Unit: 1000 people).

† The incidence rate is reported per 100 000 person-years. CI = confidence interval.

**Figure 2:**
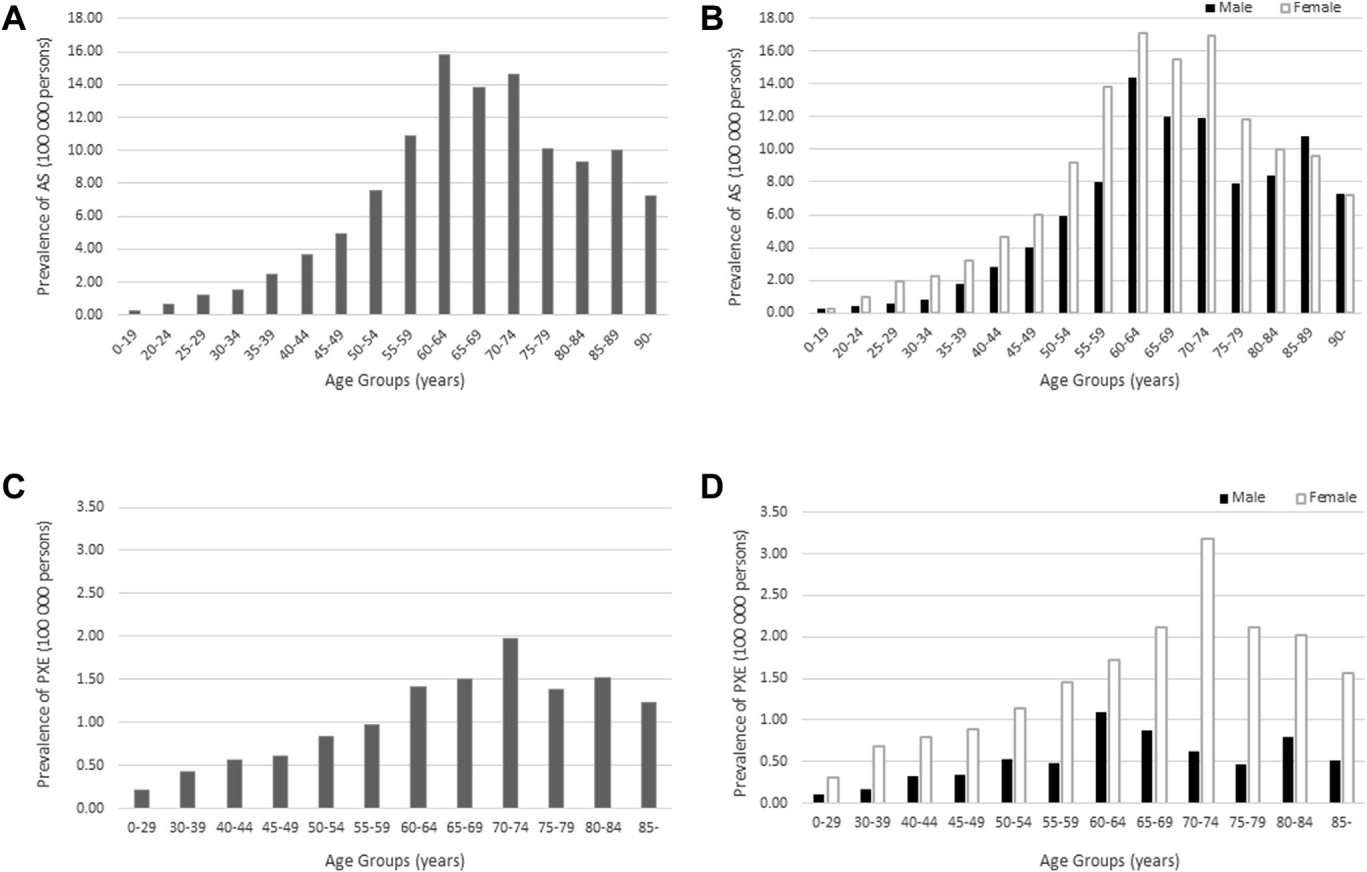
The prevalence of angioid streaks (AS) and pseudoxanthoma elasticum (PXE). The age-stratified prevalence of AS (**A**) and the age- and sex-stratified prevalence of AS (**B**) are presented. Overall, a multimodal peak was observed in the 60 to 64, 70 to 74, and 85 to 89 age groups. By sex, bimodal peaks were observed in males aged 60 to 64 and 85 to 89 years, and in females aged 60 to 64 and 70 to 74 years. The second peak occurred later in males than in females. These multimodalities may be attributed to phenotypic variation. Similarly, the age-stratified prevalence of PXE (**C**) and the age- and sex-stratified prevalence of PXE (**D**) are presented. Overall, a bimodal peak was observed in the 70 to 74 and 80 to 84 age groups. By sex, a bimodal peak was observed in males aged 60 to 64 and 80 to 84 years. In females, a peak was observed in the 70 to 74 age group.

Figure 2 and Tables 3 and 4 show the age-stratified and sex-stratified prevalence of AS and PXE. A total of 8196 cases of AS were identified in the NDB dataset on October 1, 2020, of which 61.0% were female. The prevalence of AS was 6.5 per 100 000 persons (about 1:15 000) and was higher in females than males of up to 85 years old. The highest prevalence was observed in males and females aged 60 to 64 years. A total of 1050 PXE cases were identified in the NDB dataset on October 1, 2020, of which 76.4% were female. The prevalence of PXE was 0.83 per 100 000 persons (about 1:120 000) and was higher in females than males in all age groups. The highest prevalence was observed in males aged 60 to 64 years and females aged 70 to 74 years.

**Table 3:** The Age-stratified and Sex-stratified Number of Living Patients and Prevalence of Angioid Streaks in Japan from 2011 to 2020, as Determined by the National Database of the Health Insurance Claims and Specific Health Checkups of Japan

Age Groups (Years)	Japanese Population∗			Number of Living Patients			Prevalence (95% CI)†		
	Male	Female	Total	Male	Female	Total	Male	Female	Total
0–19	10 619	10 113	20 731	25	25	50	0.24 (0.14–0.33)	0.25 (0.15–0.34)	0.24 (0.17–0.31)
20–24	3289	3081	6370	13	29	42	0.40 (0.18–0.61)	0.94 (0.60–1.28)	0.66 (0.46–0.86)
25–29	3241	3036	6277	18	58	76	0.56 (0.30–0.81)	1.91 (1.42–2.40)	1.21 (0.96–1.48)
30–34	3371	3225	6596	27	73	100	0.80 (0.50–1.10)	2.26 (1.74–2.78)	1.52 (1.22–1.81)
35–39	3756	3655	7411	67	117	184	1.78 (1.36–2.21)	3.20 (2.62–3.78)	2.48 (2.12–2.84)
40–44	4261	4157	8419	118	192	310	2.77 (2.27–3.27)	4.62 (3.97–5.27)	3.68 (3.27–4.09)
45–49	4951	4846	9797	197	290	487	3.98 (3.42–4.53)	5.98 (5.30–6.67)	4.97 (4.53–5.41)
50–54	4366	4318	8684	258	397	655	5.91 (5.19–6.63)	9.19 (8.29–10.10)	7.54 (6.96–8.12)
55–59	3932	3940	7872	315	545	860	8.01 (7.13–8.90)	13.83 (12.67–14.99)	10.92 (10.19–11.65)
60–64	3667	3764	7431	529	643	1172	14.43 (13.20–15.66)	17.08 (15.76–18.40)	15.77 (14.87–16.67)
65–69	4015	4268	8283	482	663	1145	12.00 (10.93–13.08)	15.53 (14.35–16.72)	13.82 (13.02–14.62)
70–74	4332	4846	9178	517	822	1339	11.93 (10.91–12.96)	16.96 (15.80–18.12)	14.59 (13.81–15.37)
75–79	3182	3939	7120	253	467	720	7.95 (6.97–8.93)	11.86 (10.87–12.93)	10.11 (9.37–10.85)
80–84	2248	3168	5416	189	316	505	8.41 (7.21–9.61)	9.97 (8.87–11.07)	9.32 (8.51–10.14)
85–89	1332	2403	3735	144	231	375	10.81 (9.05–12.58)	9.61 (8.37–10.85)	10.04 (9.02–11.06)
> 90	619	1813	2433	45	131	176	7.27 (5.15–9.39)	7.23 (5.99–8.46)	7.23 (6.17–8.30)
All patients	61 181	64 572	125 753	3197	4999	8196	5.23 (5.04–5.41)	7.74 (7.53–7.96)	6.52 (6.38–6.66)

∗ Japanese population data (as of October 1, 2020) were based on the Japanese Ministry of Internal Affairs and Communications. (Unit: 1000 people).

† The prevalence is reported per 100 000 persons. CI = confidence interval.

**Table 4:** The Age-stratified and Sex-stratified Number of Living Patients and Prevalence of Pseudoxanthoma Elasticum in Japan from 2011 to 2020, as Determined by the National Database of the Health Insurance Claims and Specific Health Checkups of Japan

Age Groups (Years)	Japanese Population∗			Number of Living Patients			Prevalence (95% CI)†			
	Male	Female	Total	Male	Female	Total	Male	Female	Total	
0–29	17 149	16 230	33 378	18	51	69	0.10 (0.06–0.15)	0.31 (0.23–0.40)	0.21 (0.16–0.26)	
30–39	7127	6880	14 007	12	47	59	0.17 (0.07–0.26)	0.68 (0.49–0.88)	0.42 (0.31–0.53)	
40–44	4261	4157	8419	14	33	47	0.33 (0.16–0.50)	0.79 (0.52–1.06)	0.56 (0.40–0.72)	
45–49	4951	4846	9797	17	43	60	0.34 (0.18–0.51)	0.89 (0.62–1.15)	0.61 (0.46–0.77)	
50–54	4366	4318	8684	23	49	72	0.53 (0.31–0.74)	1.13 (0.82–1.45)	0.83 (0.64–1.02)	
55–59	3932	3940	7872	19	57	76	0.48 (0.27–0.70)	1.45 (1.07–1.82)	0.97 (0.75–1.18)	
60–64	3667	3764	7431	40	65	105	1.09 (0.75–1.43)	1.73 (1.31–2.15)	1.41 (1.14–1.68)	
65–69	4015	4268	8283	35	90	125	0.87 (0.58–1.16)	2.11 (1.67–2.54)	1.51 (1.24–1.77)	
70–74	4332	4846	9178	27	154	181	0.62 (0.39–0.86)	3.18 (2.68–3.68)	1.97 (1.68–2.26)	
75–79	3182	3939	7120	15	83	98	0.47 (0.23–0.71)	2.11 (1.65–2.56)	1.38 (1.10–1.65)	
80–84	2248	3168	5416	18	64	82	0.80 (0.43–1.17)	2.02 (1.53–2.52)	1.51 (1.19–1.84)	
> 85	1951	4216	6168	10	66	76	0.51 (0.19–0.83)	1.57 (1.19–1.94)	1.23 (0.96–1.51)	
All patients	61 181	64 572	125 753	248	802	1050	0.41 (0.35–0.46)	1.24 (1.16–1.33)	0.83 (0.78–0.89)	

∗ Japanese population data (as of October 1, 2020) were based on the Japanese Ministry of Internal Affairs and Communications. (Unit: 1000 people).

† The prevalence is reported per 100 000 persons. CI = confidence interval.

**Figure 3:**
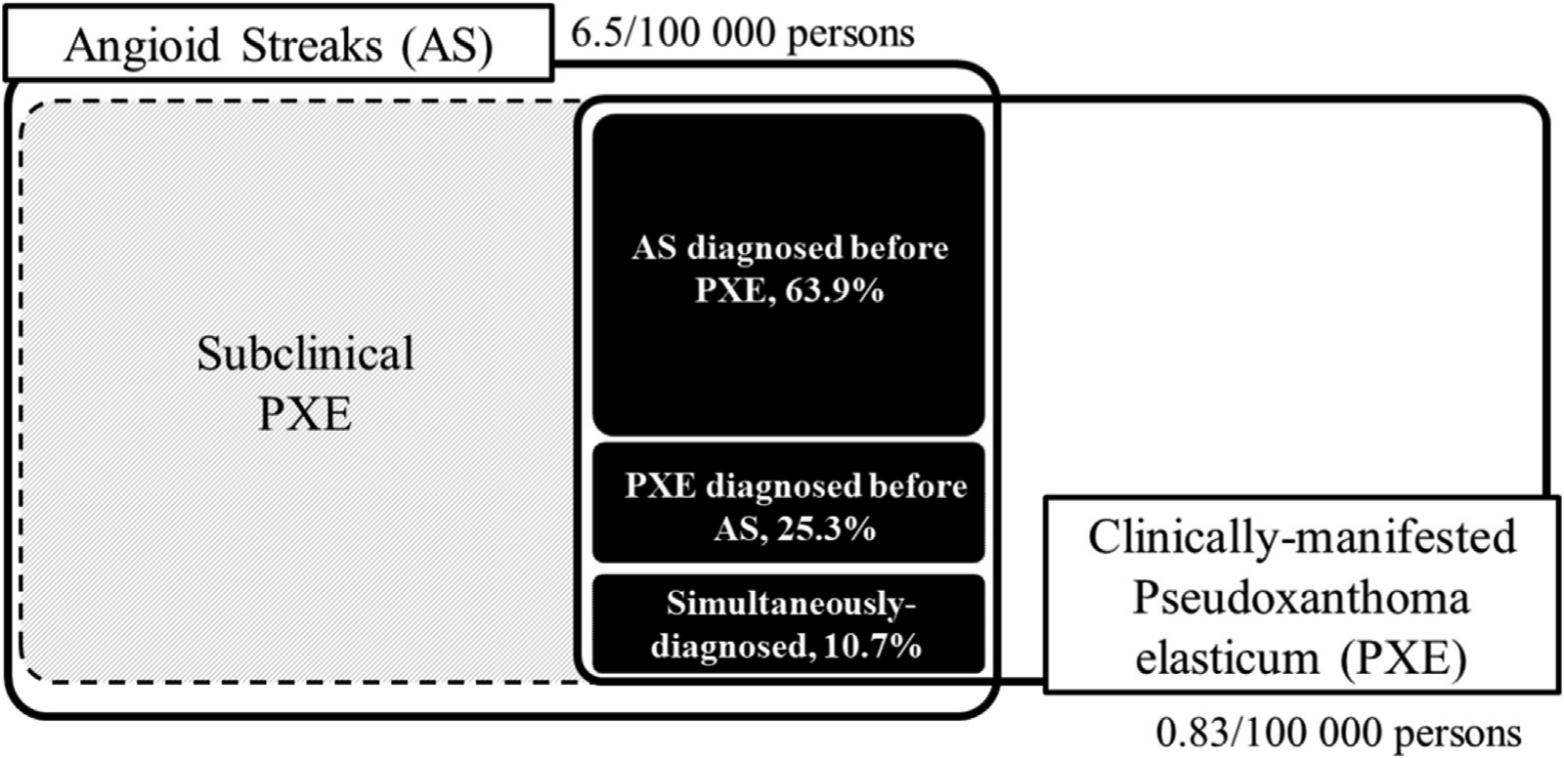
The scheme of the relationship between angioid streaks (AS) and pseudoxanthoma elasticum (PXE). The prevalence of AS and clinically manifested PXE were 6.5 and 0.83 per 100 000 persons, respectively. Among the study population, there were 363 cases of overlap between AS (8196 patients) and clinically-manifested PXE (1050 patients). Of those with overlapping conditions, 63.9% were initially diagnosed with AS, 25.3% with PXE, and the remaining 10.7% were diagnosed with both conditions simultaneously. In previous reports, skin biopsies were performed on 44 consecutive cases of AS, resulting in a diagnosis of PXE in > 90% of cases. Given these findings, the present study observed a remarkably low incidence of overlapping AS and PXE cases. These results suggest that in a substantial number of AS patients, the comorbidity of PXE may be overlooked or PXE may remain subclinical, warranting further investigation.

Figure 3 shows the overlapping of AS and PXE cases as of October 1, 2020. There were 363 patients with concurrent AS and PXE. This meant that 34.6% of 1050 patients with PXE had AS or 4.4% of 8196 patients with AS had PXE. Furthermore, 232 of the 363 patients (63.9%) with overlapping conditions were first diagnosed with AS, 92 (25.3%) were first diagnosed with PXE, and the remaining 39 (10.7%) received both diagnoses in the same month.

Four hundred and twenty-three of 6598 patients with AS and 20 of 1020 patients with PXE were confirmed dead by October 1, 2020, meaning that 6.4% of AS and 2.0% of PXE patients died during the study period. The number of deaths by age group in AS was 12 aged 50 to 59 years, 54 aged 60 to 69 years, 113 aged 70 to 79 years, 170 aged 80 to 89 years, 68 aged 90 to 99 years, and < 10 in the other age groups. In PXE, there were < 10 deaths in any age group. Table 5 shows the age of death for AS and PXE patients as of October 1, 2020. The mean age at death (± standard error) for AS and PXE was 79.3 ± 0.51 and 77.1 ± 2.68 years, respectively. Both were significantly younger (*P* < 0.00001, *P* = 0.00519) than the mean life expectancy of the general Japanese population (84.6 years).

**Table 5:** The Age of Death for Angioid Streaks and Pseudoxanthoma Elasticum Patients as of October 1, 2020

	Age of Death (Mean ± Standard Error)	Z-Score	*P* Value
Angioid streaks	79.3 ± 0.51	10.4	< 0.00001
Pseudoxanthoma elasticum	77.1 ± 2.68	2.80	0.00519
Reference (the mean life expectancy of the general Japanese population)	84.6	NA	NA

NA = not applicable.

## Discussion

The current study is the largest population-based cohort study to date to clarify the epidemiology of AS and PXE. We reported the incidence and prevalence of both diseases and observed less overlap and younger age at death compared to previous reports. This study is significant because big data analyses are especially important for rare diseases such as AS and PXE. The results of this study provide information about the current status of AS and PXE and offer the opportunity to improve the medical management of these diseases.

Although AS and PXE are clinically important diseases, there have been few reports of their prevalence. As for incidence, we found no reports in PubMed (as of November 7, 2022). Of the reports on prevalence, only 1 article by Pelttari et al^30^ used actual measurements while other studies used estimates based on formulas. Pelttari's study was a nationwide registry search using ICD-10 codes for the Finnish population through the end of 2018, and the prevalence of PXE in Finland was reported to be 1:260 000 (21 patients in a Finnish population of 5 517 919). Because both the Finish report and ours reported a prevalence in the range of 1 per 100 000 to 300 000 population, we can conclude that the prevalence of PXE is within this range. The multimodal peaks observed in the age-stratified and sex-stratified prevalence and incidence of AS and PXE may reveal the phenotypic variabilities of these diseases.

While a previous study reported that most patients with AS have PXE,^2^ in the current study, the overlapping of PXE was 4.4% which was far lower than expected. The reason may be the difference in diagnostic strategies for PXE between the actual clinical and clinical studies. In our previous report, we performed skin biopsies for consecutive AS with or without skin lesions, leading to the diagnosis of nearly 90% as PXE.^5^ However, in actual clinical practice, we do not usually perform a skin biopsy if no skin lesions are present. Germain^9^ also reported that histologic findings of PXE may be present even in the absence of gross skin findings. The current results showed the important fact that the majority of PXE may remain subclinical and undetected.

Even when subclinical, diagnosing PXE is important. As mentioned earlier, PXE is considered a life-threatening disease associated with ischemic heart disease, cerebral infarction, and gastrointestinal bleeding, and carries a risk of sudden death.^31^ According to the MHLW survey, screening by a cardiologist to detect ischemic heart disease and screening with upper gastrointestinal endoscopy or contrast-enhanced computed tomography to detect gastrointestinal bleeding are recommended.^15^ Early detection and treatment of these potentially fatal complications in patients with PXE is important and may lead to improvement in the lives of patients with PXE. In this study, we not only confirmed that the age of death of PXE patients (77.1 ± 2.68 years) was significantly lower (*P* = 0.00519) than the mean life expectancy in Japan (84.6 years as a whole) (available at https://databank.worldbank.org/reports.aspx?source=2&series=SP.DYN.LE00.IN&country=JPN, accessed February 24, 2023) but also clarified that the age of death of AS patients (79.3 ± 0.51 years) was significantly lower (*P* < 0.00001) than the mean life expectancy in Japan. Despite AS being explicitly comorbid with PXE in only 4.4%, patient age of death was almost similar to that of PXE. Although it is not possible to directly prove from the current data whether premature death in AS patients is caused by the presence of PXE, based on previous reports indicating a high rate of coexistence between AS and subclinical PXE, we speculate that premature death in AS patients can be attributed to the coexistence of subclinical PXE. Thus, we believe, adherence to guidelines,^15^ instructing ophthalmologists to consult dermatologists and internal medicine specialists and intervene therapeutically at an early stage following AS detection should also be emphasized.

The study has several limitations that warrant caution in interpreting the results. First, although the NDB covers medical care for almost the entire Japanese population, the true prevalence and incidence of AS and PXE may be underestimated because medical care provided by social welfare, automobile liability insurance, and workers' compensation insurance is not included in the NDB.^17^^,^^18^^,^^32^ However, since such cases are uncommon, we believe that their influence is minimal. Second, the diagnosis of AS and PXE based on NDB diagnostic codes is not perfect because some patients may have been given other alternative diagnoses. Ideally, this study should be conducted after verifying the correctness of the diagnosis by diagnostic codes. However, AS and PXE are less likely to be misdiagnosed because they are typical clinical conditions. Therefore, we consider that there will be little impact on the results of this study. Third, we were unable to identify AS and PXE in those who did not visit a hospital. This could lead to an underestimation of the incidence and prevalence of AS and PXE. However, a smaller database than our study, such as hospital-based data, would not be able to count patients of other hospitals either, leading to a more significant underestimation. Therefore, we believe that our database analysis is the most appropriate and feasible method. Finally, the interpretation of death must be considered because the death flags in the claims are not perfect. However, as its specificity is reported to be almost 100%, patients treated as a death in the current study would have truly died at the time of the death flag.^33^ Furthermore, sensitivity is reported to be about 80%^33^ meaning that only approximately 1% error is made in the prevalence calculation. Thus, we believe our analysis is reasonably reliable.

In conclusion, we have examined the epidemiology of AS and PXE, 2 intractable and rare diseases, using the NDB database, which is the largest in terms of size and comprehensive coverage. Although there are some limitations, we have obtained valuable data with evidence. Especially because subclinical PXE can have as high a mortality rate as clinically-manifested PXE, patients with AS should see dermatologists even if they do not have skin lesions.
